# Efficacy of an intranasally administered live attenuated PRRSV-2 vaccine against challenge with a highly virulent PRRSV-1 strain

**DOI:** 10.3389/fvets.2025.1619052

**Published:** 2025-08-22

**Authors:** E. Mateu, M. Cortey, M. S. Serena, I. Domingo-Carreño, M. Alberch, L. Aguirre, I. Diaz, M. Martín, J. M. Sanchez-Carvajal, I. M. Rodriguez-Gomez, J. H. Lara-Puente, C. Artigas-Cabre, D. Sarfati-Mizrahi, B. Lozano-Dubernard

**Affiliations:** ^1^Dept. Sanitat i d’Anatomia Animals, Facultat de Veterinària, Universitat Autònoma de Barcelona, Barcelona, Spain; ^2^Dept. Anatomía y Anatomía Patológica Comparadas, Universidad de Córdoba, Córdoba, Spain; ^3^Laboratorio Avi-Mex, S. A. de C. V., Ciudad de Mexico, Mexico; ^4^Independent Researcher, Barcelona, Spain

**Keywords:** porcine reproductive and respiratory syndrome virus, intranasal vaccine, immunity, virulence, clinical signs, lung pathology

## Abstract

**Introduction:**

The emergence of highly virulent strains of the porcine reproductive and respiratory syndrome virus has driven the need for new vaccines. This study evaluates the efficacy of an intranasal (IN) vaccine composed of a naturally attenuated PRRSV-2 isolate, compared to a commercially available intramuscularly administered (IM) PRRSV-1 vaccine, against a heterologous challenge with a highly virulent PRRSV-1 strain (R1).

**Methods:**

Sixty-eight PRRSV-naïve pigs were divided into four groups: two non-vaccinated controls (NV/NCh, NV/Ch), one IM-vaccinated with a PRRSV-1 MLV (Por), and one intranasally (IN)-vaccinated with the PRRSV-2 vaccine (IL).

**Results:**

Clinical, pathological, and immunological outcomes were assessed post-challenge. Both vaccines significantly (*p* < 0.05) reduced fever duration (3–5 days less than controls, respectively), reduced the clinical scores after challenge, and mitigated weight loss (*p* < 0.05), though viral loads in serum and lungs remained comparable across groups. Macroscopic lung lesions at 10 days post-challenge (DPC) were reduced in vaccinated groups (13–14% of pneumonic lung on average in vaccinated groups vs. 35% in NV/Ch), yet microscopic lesions persisted, correlating with lung viral loads at 28 DPC (*R*^2^ = 0.54, *p* < 0.001). None of the tested vaccines achieved an efficient control of the viremia or nasal shedding compared to unvaccinated controls. Cross-reactive cell-mediated responses suggested shared epitopes between PRRSV-1 and PRRSV-2; however, the frequencies of interferon-gamma-secreting cells did not correlate with lesion severity.

**Discussion:**

The IN vaccine demonstrated non-inferiority to IM vaccination in alleviating clinical signs and helped reduce weight losses, however, at later times control of viral replication was lower, underscoring limitations in heterologous protection. The dissociation between systemic immune markers and tissue-specific outcomes highlights the need for strategies targeting tissue-resident immunity. These findings advocate further exploration of mucosal vaccination as a complementary strategy for PRRSV control, particularly under heterologous challenge conditions, while emphasizing the persistent challenges posed by viral diversity and incomplete cross-protection.

## 1 Introduction

More than 30 years after its emergence, the *Porcine reproductive and respiratory syndrome virus* (PRRSV) remains one of the leading causes of economic losses and mortality in the global swine industry. For both PRRSV species, PRRSV-1 and PRRSV-2, infection of pregnant sows has been shown to cause abortions, fetal mummifications and stillbirths (1). Furthermore, infection of sows in late pregnancy can result in vertical transmission and the birth of viremic piglets that may remain viremic for months, thereby serving as sources of infection within the herd (2). Similarly, both PRRSV-1 and PRRSV-2 are considered major contributors to the porcine respiratory complex in nurseries and fattening units, favoring secondary infections and acting synergistically with other pathogens (3). The emergence of highly virulent PRRSV strains has further worsened the picture. Recent examples include the emergence of L1C PRRSV-2 strains in the USA (4, 5), or the highly virulent PRRSV-1 in Spain (6, 7), where mortality rates are significantly higher and can affect sows as well. In such cases, the severity of lung lesions is very high and interstitial, bronchointerstial and proliferative necrotizing pneumonia may be observed in the same affected individual (8, 9).

In pig-dense areas, PRRSV control is difficult because of the high probability of contact with infected animals, contaminated fomites or airborne. This requires the implementation of advanced biosecurity measures to reduce the risk of infection (10). Additionally, the genetic and antigenic diversity of the virus further complicates control efforts. In practical terms, this diversity implies that after infection the heterologous immunity is only partial (11, 12). However, vaccines are a useful tool in the reduction of the consequences of infection. Thus, vaccinated sows are significantly protected against the reproductive disease, although they can still transmit the infection to their offspring (13, 14). In the respiratory model, vaccination has been shown to reduce the severity and extent of lung lesions (15). In both the reproductive and respiratory models, vaccination has been shown to reduce the duration of viremia. Several commercial vaccines against PRRSV-1 and PRRSV-2 are available. Modified live vaccines (MLVs) are generally preferred due to their ability to elicit a more robust immune response, whereas inactivated vaccines are considered less efficacious (16). Notably, cross-protection between the two PRRSV species is limited; however, some evidence suggests that vaccines targeting PRRSV-2 may provide better protection against PRRSV-1 than vice versa (17, 18). Other types of vaccines against PRRSV based on new technologies such as mRNA (19) or nanoparticles (20) are currently being explored, although they have not yet reached commercial development [reviewed by He et al. (21)].

Commercial PRRSV vaccines are generally designed for intramuscular or intradermal administration. The intranasal use of PRRSV vaccines has been little studied. However, the available evidence for MLV suggests that it is at least as effective as intramuscular vaccines (22). Since intranasal administration is expected to enhance mucosal immunity at the portal of entry, it may be a promising strategy for reducing the respiratory impact of highly virulent PRRSV strains.

In this study, we assessed the efficacy of a PRRSV-2 live vaccine delivered intranasally (G16X/Innovac L-PRRS, a naturally highly attenuated strain) against the challenge with a highly virulent PRRSV-1 isolate in comparison with an intramuscularly administered commercial PRRSV-1 vaccine.

## 2 Materials and methods

### 2.1 Design of the study

**Figure 1:**
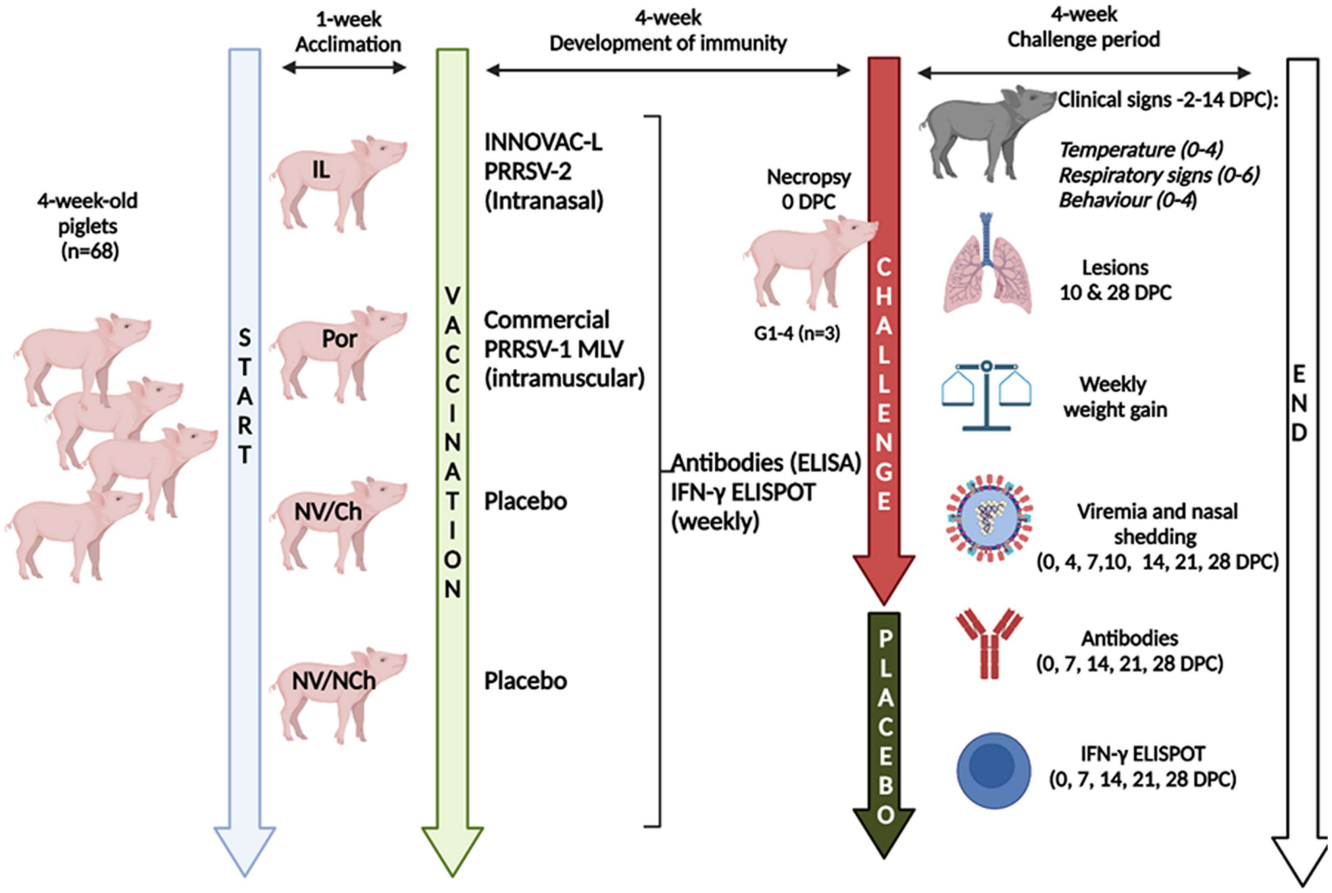
Graphical summary of the design. V/NCh, non-vaccinated/non-challenged; NV/Ch, non-vaccinated/challenged; Por, Porcilis PRRS vaccinated/challenged; IL, Innovac L vaccinated/challenged. Created in BioRender. Mateu, E. (2025) https://www.biorender.com/8dste86.

The study included four groups of animals: non-vaccinated/non-challenged (NV/NCh); non-vaccinated/challenged (NV/Ch); intramuscularly vaccinated with a commercial (Porcilis® PRRS) PRRSV-1 MLV (Por); and a group of pigs intranasally vaccinated with a commercial (Innovac-L®) PRRSV-2 vaccine (IL). Vaccines were administered according to the manufacturer’s directions. Figure 1 summarizes the design. Briefly, 68 4-week-old Landrace x Duroc pigs were purchased from a historically PRRSV-free farm (confirmed by ELISA and RT-qPCR using the same commercial kits as those used during the vaccination and challenges phases, as explained below) and transported to experimental facilities. Upon arrival, animals were ear-tagged and randomly assigned to four groups of 17 pigs each that were physically separated in different rooms with no direct contact between them. They were allowed to acclimate for 1 week, after which they receive 2.0 mL of sterile saline intramuscularly as a placebo (NV/NCh; NV/Ch), or were intramuscularly vaccinated (2.0 mL) with the PRRSV-1 MLV according to the manufacturer’s instructions; intranasally vaccinated with the PRRSV-2 vaccine (1.0 mL/nostril using an intranasal mucosal atomization device, MAD Nasal ™). Five weeks later, 3 animals per group were euthanized to assess lung lesions before challenge in the vaccinated and control animals. Then, the remaining pigs in groups NV/Ch, Por and IL were intranasally challenged (1.0 mL/nostril administered with the atomization device; 10^5.5^ TCID_50_/ml) with the strain R1 (Genbank accession number OM893828), belonging to a highly virulent clade recently described in Spain (6) and produced as a passage 4 in porcine alveolar macrophages (PAMs). During the challenge phase the isolation of animals in separated rooms was maintained. PAMs were tested by PCR and confirmed negative for PRRSV, porcine circovirus 2 and mycoplasma before the production of the inoculum. Ten days post-challenge, 7 animals/groups were euthanized to assess lung lesions. The experiment ended at 28 days post-challenge (DPC) with the necropsy of the remaining animals.

### 2.2 Sampling

Blood samples were collected weekly via jugular venipuncture from the day of vaccination until the end of the experiment. Additionally, samples were taken on days 4 and 10 post-challenge. Blood was collected either in regular siliconized tubes for serum preparation or in heparinized tubes for the isolation of of peripheral blood mononuclear cells (PBMC). Sera were aliquoted and stored at −80 °C until needed.

After the challenge and until the end of the experiment, nasal swab (Virocult®) samples were taken on days 0, 4, 7, 10, 14, 21 and 28 post-challenge. Nasal samples were collected by inserting the swabs into both nostrils. Then, swabs were manually resuspended in transport medium and transported to the laboratory at 4 °C. Upon arrival, the tubes containing the swabs and medium were vigorously vortexed to achieve effective resuspension of the collected material, centrifuged at 500 g for 5 min at 4 °C and the supernatant was stored in aliquots at −80 °C.

On the days that necropsies were performed (10 and 28 DPC), samples were collected from the cranial, middle, and right diaphragmatic lobes of each animal, both fresh and fixed in buffered formalin.

### 2.3 Clinical scores

Body temperature and clinical signs were monitored from 2 days before the challenge until 14 days post-challenge (DPC). Temperatures were scored on a scale 0–4 scale as follows: 0 ≤ 40.0 °C; 1 = 40.01–40.50 °C; 2 = 40.51–41.00 °C; 3 = 41.01–41.50 °C; 4 ≥ 41.51.

Respiratory signs were recorded in a 0–6 scale, where 0 = no abnormal signs; 1 = clear nasal discharge with no other signs, 2 = sneezing, 3 = cough with normal breathing, 4 = slightly difficult breathing with or without cough, 5 = clear difficult breathing; 6 = gasping.

Finally, behavioral changes were scored according to the following scale: 0 = normal; 1 = apathetic but stands when stimulated; 2 = prostrated but responds when touched, 3 = prostrated, conscient but unresponsive to be touched, 4 = unconscious. For each animal, a final clinical score was calculated by summing the three aforementioned scores. A proportional aggregate value was calculated as the total score for a group on a given day divided by the maximum possible score for that group and day, multiplied by 100 ([*Σ* scores observed in the group/Σ maximum possible score for the group and day]*100). So, a score of 100 would mean maximum clinical scores in all categories for all animals for a given group.

### 2.4 Weight gains

Animals were weighed upon arrival, on the day of the challenge and, subsequently, on a weekly basis until the end of the experiment. Individual weight gain was calculated by subtracting the initial weight from the final weight for each observation period.

### 2.5 Macroscopic and microscopic lung lesion scoring

On days 0 (*n* = 3 per group), 10 (*n* = 7 per group) and 28 post-challenge (*n* = 7 per group), the animals designated for this purpose were euthanized by an anesthetic overdose. Animals selected for euthanasia were chosen using a randomized allocation system. Macroscopic lesions were assessed visually according to Halbur et al. (23). Briefly, in this scheme the total lung was assigned a value of 100, with each lobe (dorsal and ventral views) allocated a proportional score.

Thus, in the dorsal view, the cranial, and middle lobes were assigned a value of 5 and the diaphragmatic lobes a value of 15; the maximum score for the dorsal view was therefore 50 (5 + 5 + 5 right and left (cranial and caudal parts) cranial lobes; 5 left middle lobes and, 15 + 15 right and left diaphragmatic lobes); in the ventral view, the cranial and middle lobes were again assigned a value of 5, the diaphragmatic lobes a value of 12.5 and the accessory lobe a value of 5 (50 points for the ventral view). A score was assigned to each lobe based on the affected lung area. For example, if the dorsal view gave a score of 2.5 for the cranial lobe, this indicated that lung lesions were observed in 50% of that lobe. Additional lung abnormalities (e.g., fibrin, hemorrhage) were noted on individual record sheets.

The histopathological evaluation included the assessment of the severity of interstitial pneumonia (0–4; 0 = no lesion, 1 = mild; 2 = moderate multifocal, 3 = moderate diffuse, 4 = severe), bronchopneumonia (0–4; 0 = no lesion, 1 = mild, 2 = moderate multifocal; 3 = moderate diffuse, 4 = severe), and the presence or absence of proliferative necrotizing pneumonia (0–1), resulting in a maximum score of 9. Tissue sections (4 μm thick) were stained with hematoxylin and eosin (H&E) and blindly evaluated by two experienced pathologists and the mean score for each sample was reported.

### 2.6 Viremia, nasal shedding and viral load in lungs

A commercial RT-qPCR kit (LSI VETMAX PRRS EU & NA 2.0, Thermofisher) was used to determine to detect the virus in serum, nasal swabs, and lung samples. The kit allowed a differential detection of PRRSV-1 and PRRSV-2. For lungs, a piece of 1 cm3 was excised from each left lung lobe, weighed, grinded and resuspended in sterile PBS (1 mL PBS/g of tissue). Samples were then centrifuged, and the supernatant was used for the extraction. RNA extraction from the samples was carried out using the MagMax Core Nucleic Acid Purification kit (Thermofisher) and a KingFisher Flex robot (Thermofisher).

Additionally, all RT-qPCR positive samples from days 0, 21 and 28 post-challenge were inoculated onto PAMs and MARC-145 cell cultures for the isolation of the virus. Viral isolation was confirmed by cytopathic effects and immunofluorescent staining a with monoclonal antibody 1CH5, specific for the PRRSV-1 nucleoprotein, and a FITC-conjugate anti mouse IgG1° antibody.

For the calculation of viral loads, decimal dilutions of the challenge virus were tested in triplicate in the RT-qPCR assay and results were used in linear regression analysis. The resulting formula was used to extrapolate the viral loads in TCID_50_/ml from the Ct values (Supplementary material 1 shows the regression results).

### 2.7 Antibody detection

Antibody responses against PRRSV were assessed using the IDEXX PRRS X3 Ab kit, which detects antibodies produced against the nucleoprotein of PRRSV. This kit detects antibodies against both PRRSV-1 and PRRSV-2.

### 2.8 IFN-*γ* ELISPOT

Virus-specific interferon-gamma-secreting cells (IFN-*γ*-SC) were quantified using the ELISPOT assay. For this purpose, PBMC were separated from heparinized blood samples by gradient centrifugation using Histopaque 1.077 (Merck). PVDF plates (MultiScreen® HTS IP, Merck) were coated with the capture antibody against IFN-γ (clone P2G10, BD Pharmingen) at a concentration of 10 μg/mL in PBS. Prior to coating, the plates were pretreated with 35% ethanol for 30 s. After incubating the plates at 4 °C overnight, PBMCs were added (2.5×10^5^ viable cells/well) and stimulated with Porcilis strain, Innovac strain or R1 strain at a multiplicity of infection of 0.1, depending on the groups and whether it was the immunization or the challenge phase. Unstimulated wells (containing only culture medium) served as background controls, while Concanavalin A (10 μg/mL) was used as a positive control. After 20 h of incubation, cells were removed, plates were washed with PBS and the biotinylated detection antibody P2C11 (BD Pharmingen) was added at a concentration of 0.75 μg/mL. After 2 h of incubation at room temperature, plates were washed, and streptavidin-peroxidase (Merck, 0.67 μg/mL) was added and incubated for 1 h at room temperature. Then, unbound streptavidin was removed by washing with PBS and then, the AEC substrate (BD Pharmingen) was added to reveal the reaction. Spot formation was assessed using stereoscopic examination. Virus-specific IFN-*γ*-SC counts were obtained by subtracting the mean number of spots/well in the unstimulated wells from the counts of stimulated wells. The wells in which concanavalin A (Merck) had been added were used as positive controls indicating cell functionality. Results were reported as the number of virus-specific IFN-γ-SC per million PBMCs.

### 2.9 Statistical analysis

Statistical analyses were performed using GraphPad Prism v10.2.3. For quantitative variables, between-group comparisons were made using the Mann–Whitney method (two-group comparison) or the Kruskal-Wallis method with Dunn’s correction for multiple comparisons (more than two groups). For qualitative variables, the chi-square calculation was used, using Fisher’s exact *p*-values where necessary. Correlations between viral loads and average lesion score were performed using linear regression analyses. The area under the curve (AUC) was calculated for viral load measurements.

## 3 Results

### 3.1 Clinical scoring

**Figure 2:**
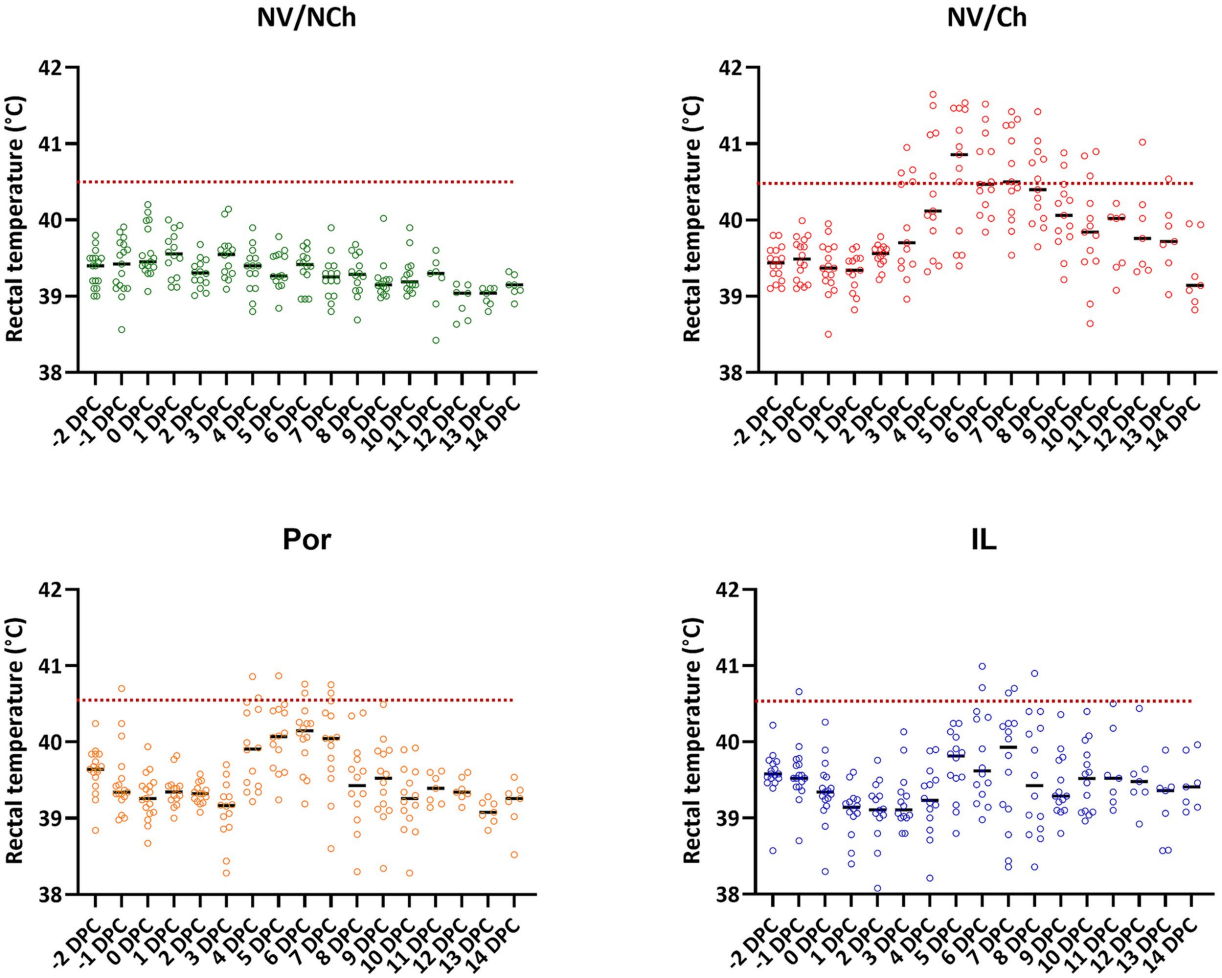
Individual rectal temperature recordings for the different groups. The graphs show the distribution of individual temperature recordings in each group. Dotted lines indicate 40.5 °C. NV/NCh, non-vaccinated/non-challenged; NV/Ch, non-vaccinated/challenged; Por, Porcilis PRRS vaccinated/challenged; IL, Innovac L vaccinated/challenged.

At the time of challenge all animals appeared clinically healthy. On that day, the rectal temperatures recorded were within the normal range considering the age and handling of the animals. Figure 2 shows the rectal temperature results for each group. As the graphs show, the rectal temperature of the NV/Ch animals started to rise from day 3 post-challenge, with temperatures above 40.5 °C being recorded consecutively in this group until 10 DPC. In the Por group rectal temperatures above 40.5 °C were observed only between days 3 and 7 post-challenge, namely, 3 days less than in the NV/Ch. In the IL group, temperatures above 40.5 °C were only recorded between day 6 and 8 post-challenge, that is 5 days less than in the NV/Ch and 2 days less than in the Por group.

Detailed examination of fever development by days of observation showed significant differences in the mean temperatures recorded in the NV/Ch group compared to the IL group for days 2 to 9 post-challenge (Supplementary material 2). Between the two vaccinated groups, significant differences were only observed on day 4 post-challenge, in favor of IL.

In the NV/Ch group, 25.9% of temperature readings during the challenge period were at or above 40.5 °C. This proportion was significantly higher (*p* < 0.01) than that recorded for Por (4.3%) or IL groups (3.2%).

**Figure 3:**
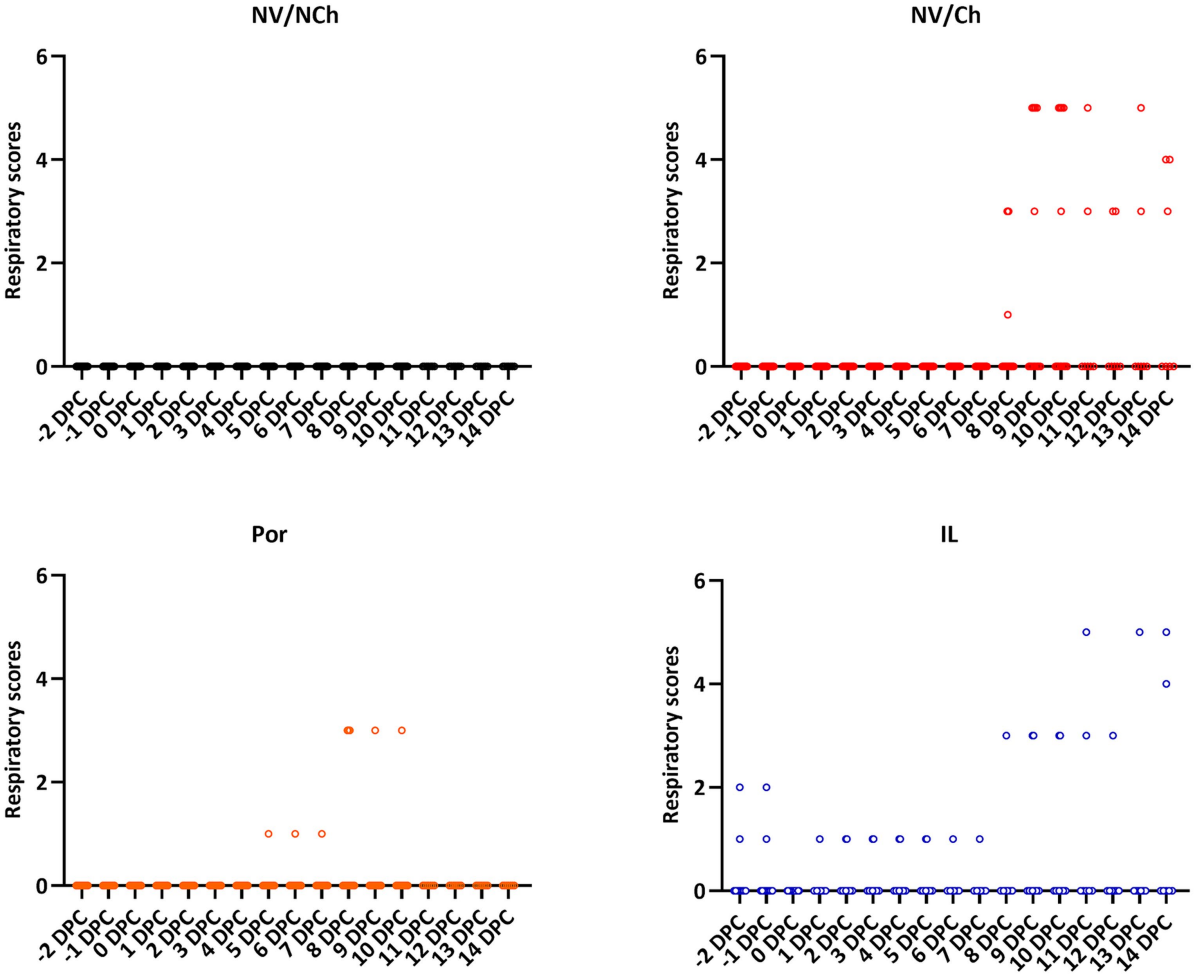
Individual respiratory scores for the different groups and days. The graphs show the distribution of individual clinical scores (X-axis = days after challenge; Y-axis = respiratory scores). NV/NCh, non-vaccinated/non-challenged; NV/Ch, non-vaccinated/challenged; Por, Porcilis PRRS vaccinated/challenged; IL, Innovac L vaccinated/challenged.

Regarding the development of clinical signs, respiratory signs were evident in the NV/Ch from 8 DPC, extending throughout the rest of the observation period (Figure 3). Respiratory signs included coughing and labored breathing. In contrast, in vaccinated groups (Por and IL) less severe signs were observed, mostly nasal discharge, although in group IL occasionally more serious signs were observed from day 10 post-challenge. Regarding the behavioral data, feverish animals were apathetic (score 1) and only NV/Ch showed lethargy (score 2 or higher).

**Figure 4:**
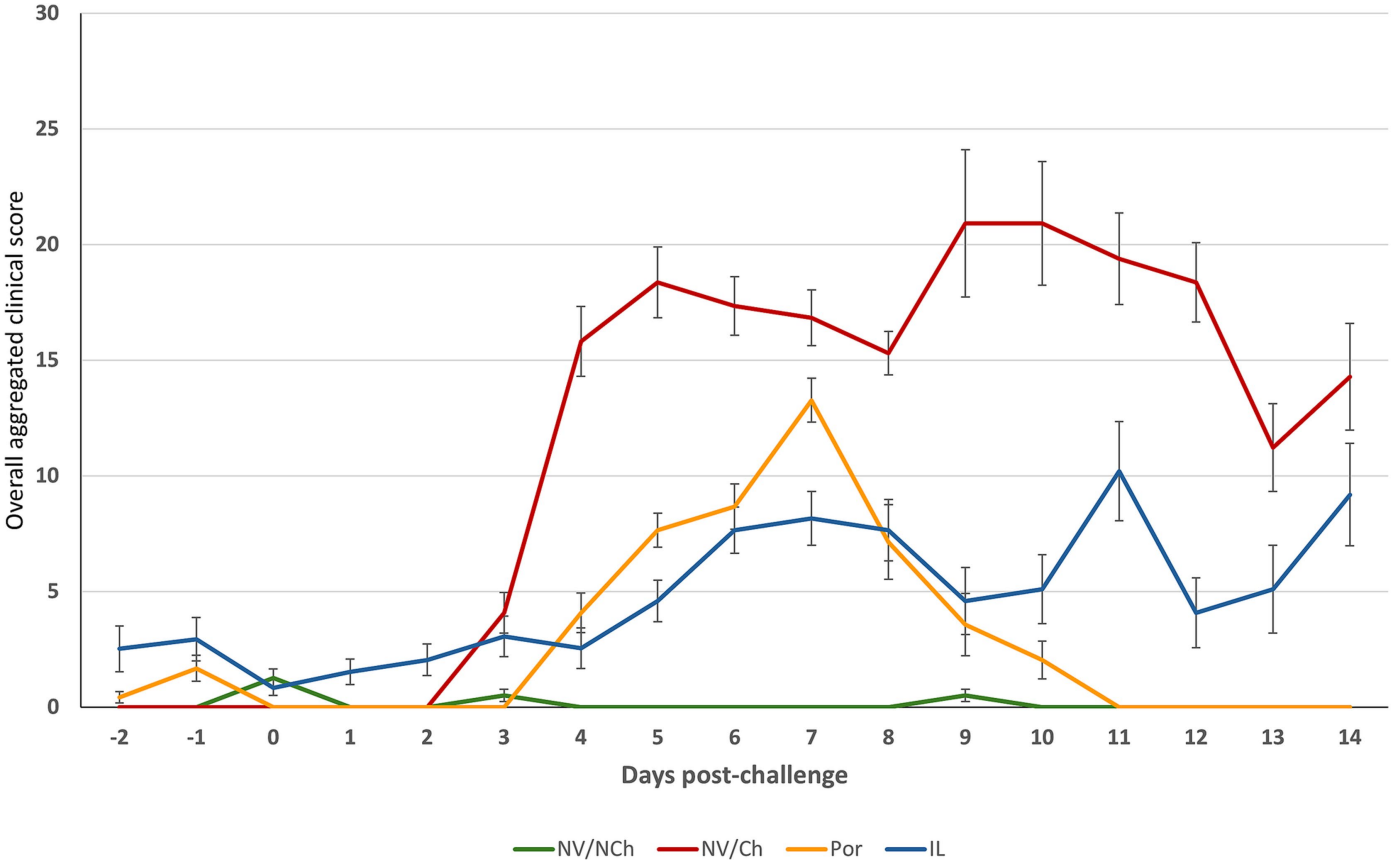
Overall clinical scores throughout the post-challenge observation period. The graphs show the average overall clinical score. (X-axis = days after challenge; Y-axis = overall average clinical scores). NV/NCh, non-vaccinated/non-challenged; NV/Ch, non-vaccinated/challenged; Por, Porcilis PRRS vaccinated/challenged; IL, Innovac L vaccinated/challenged. Bars show the standard deviation for the sum of scores for each group and day.

An overall aggregated clinical score was calculated from the recorded clinical data (Figure 4). It showed a reduction in severity in the vaccinated animals compared to the non-vaccinated/challenged controls. Daily clinical scores (see Supplementary material 3) revealed significant differences favoring the vaccinated groups (*p* < 0.05) on days 4–6, 9–10 and 12 post-challenge. Overall scores for the two vaccinated groups were similar for most of the days.

### 3.2 Weight gains

**Figure 5:**
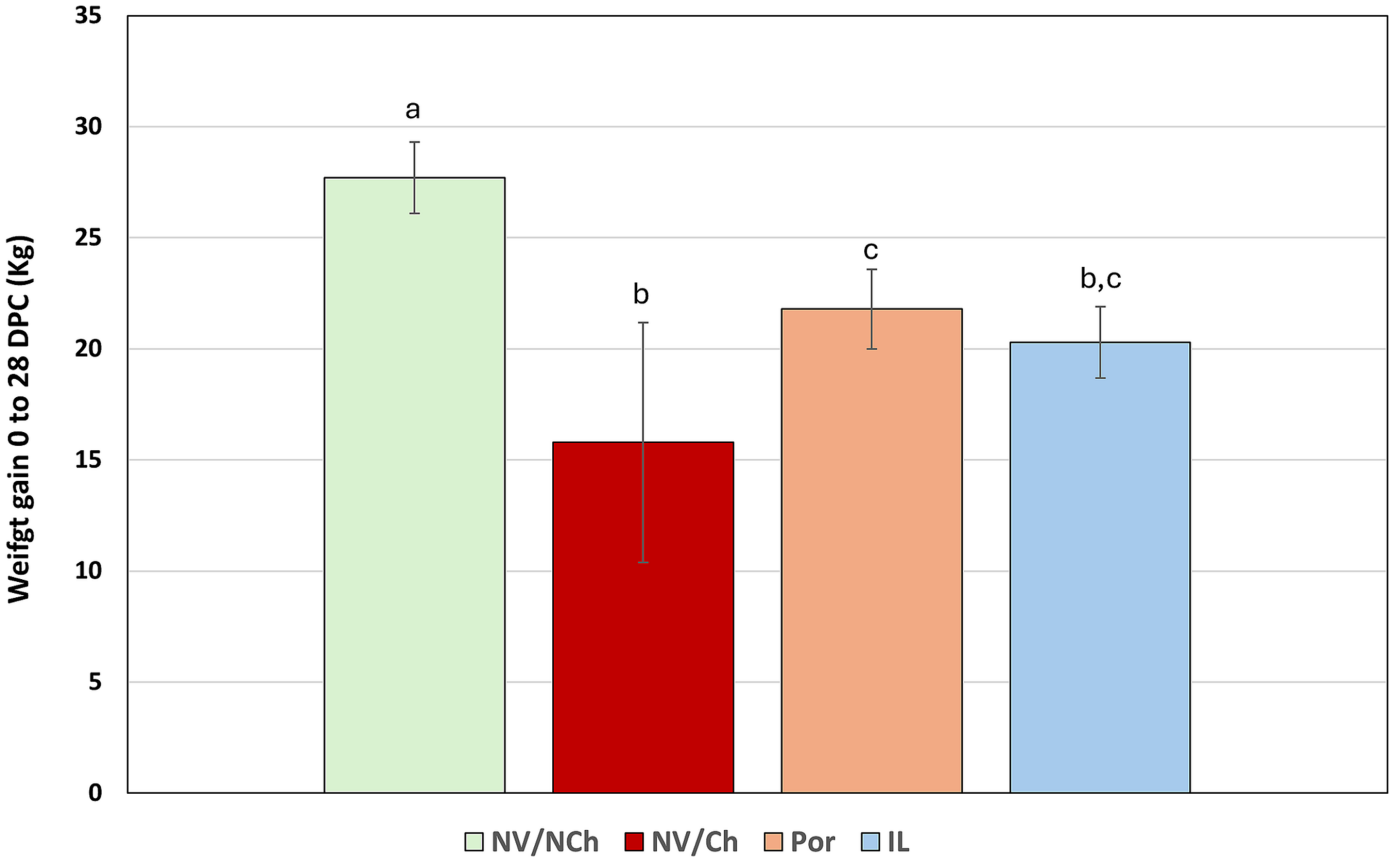
Average weight gains (kg) for the whole challenge phase [0–28 days post challenge (DPC)]. The graph shows the mean and standard deviation for total weight gain between day 0 and 28 post-challenge in each group. Superscripts with different letters indicate statistically significant differences (*p* < 0.05). NV/NCh, non-vaccinated/non-challenged; NV/Ch, non-vaccinated/challenged; Por, Porcilis PRRS vaccinated/challenged; IL, Innovac L vaccinated/challenged. Bars show the standard deviation.

Analysis of weight gain during the challenge period revealed significant differences between groups (p < 0.05). As shown in Figure 5, NV/NCh controls gained an average 27.7 kg (989.3 g/day) while the NV/Ch group gained only 15.79 kg (563.92 g/day) (*p* < 0.05). The vaccinated groups gained 21.79 kg (778.21 g/day) and 20.29 kg (724.46 g/day) for Por and IL, respectively. Notably, the greatest reduction in weight gain occurred during the second and third weeks post-challenge, although some animals already suffered heavy weight losses in the first week post-challenge. Weekly weight gains per group are shown in Supplementary material 4.

### 3.3 Lung lesions

Lung lesions were assessed both macroscopically (percentage of affected lungs) and microscopically (severity of lesions). The first assessments were performed at the time of challenge with the aim of determining possible vaccine-induced lesions. At 0 DPC, in vaccinated animals, macroscopic pneumonia scores ranged from 0 to 4% of the lung (4, 1.5 and 0% for the Por group and, 0, 0 and 1% in the IL group).

**Figure 6:**
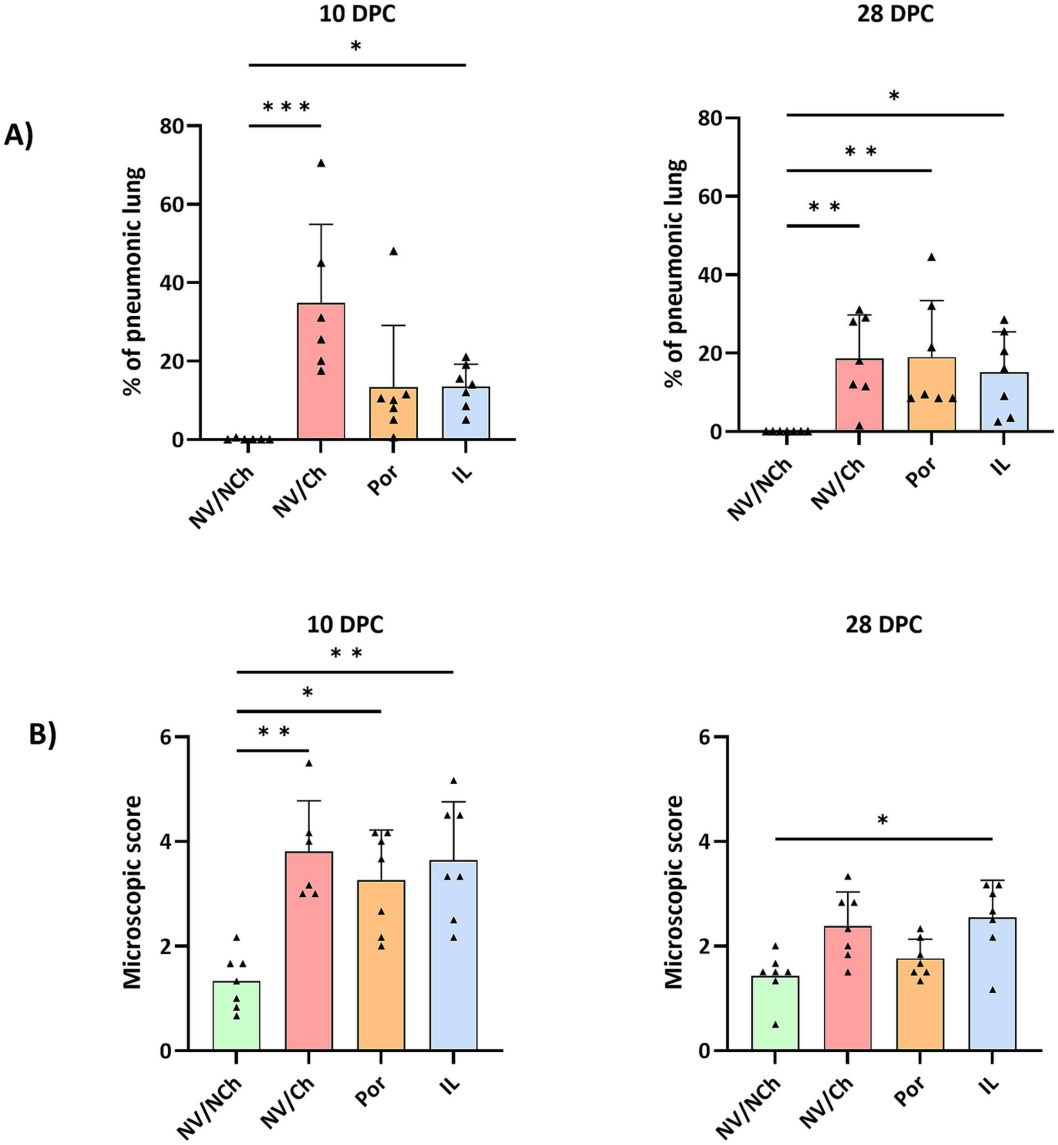
Lung lesions. The figure depicts the distribution of individual scores (triangles), the average (bars), and the standard deviation (error bars) for the macroscopic assessment of pneumonia **(A)** and the microscopic lesions **(B)** at 10- and 28DPC. NV/NCh, non-vaccinated/non-challenged; NV/Ch, non-vaccinated/challenged; Por, Porcilis PRRS vaccinated/challenged; IL, Innovac L vaccinated/challenged. *, *p* < 0.05; **, *p* < 0.01; ***, *p* < 0.001.

At 10 DPC, extensive lesions were observed in the challenged groups that scored up to 70% of the affected lung (Figure 6). The mean percentage of affected lung tissue was 34.9 ± 20.0% in NV/Ch, 13.4 ± 15.7% in Por, and 13.6 ± 5.6% in IL animals. At 28 DPC, these values were 18.7 ± 11.1%; 19.0 ± 14.4% and 15.1 ± 10.4% for NV/Ch, Por and IL groups, respectively, indicating the persistence of large areas of affected lung. Regarding the microscopic score indicating the severity of the lesions, at 10 DPC, average scores were 3.8 ± 1.0; 3.3 ± 0.9 and 3.6 ± 1.1 for NV/Ch, Por and IL groups, respectively. At 28 DPC those scores were significantly reduced to 2.4 ± 0.7; 1.7 ± 0.4 and 2.5 ± 0.7 for the same groups.

**Figure 7:**
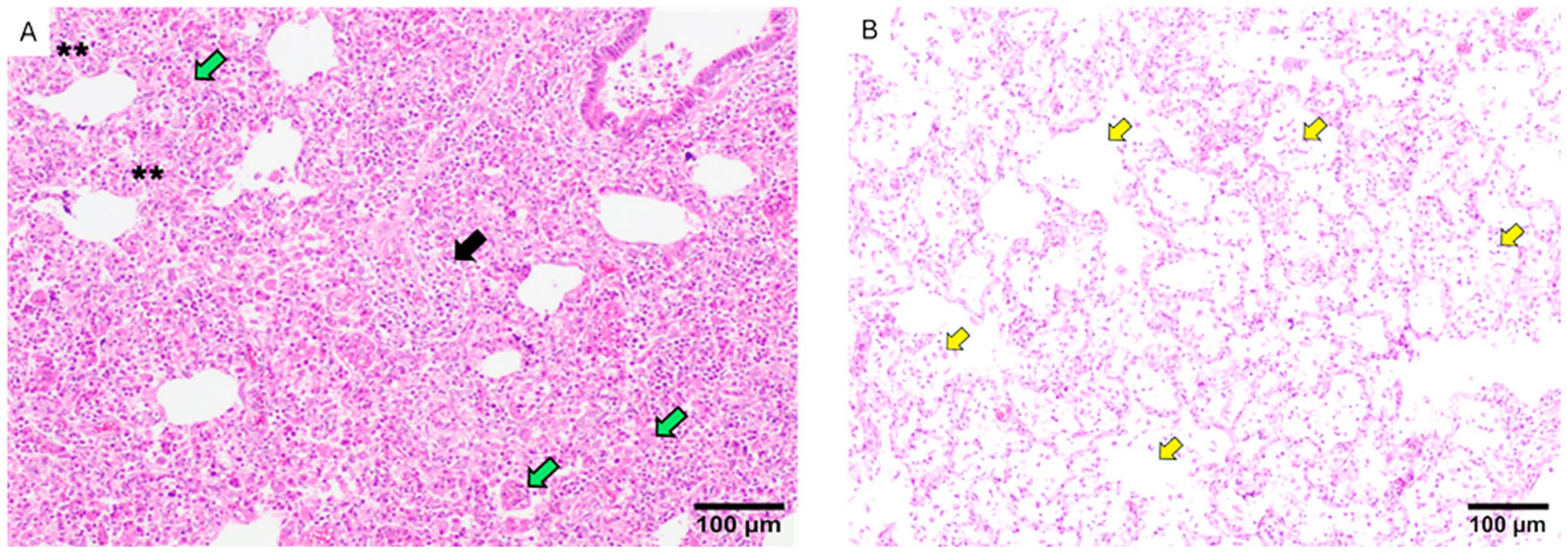
Examples of lung lesions observed in histopathological analyses. **(A)** The figure shows areas of severe interstitial pneumonia (two asterisks), foci of proliferative necrotizing proliferative pneumonia (green arrows) and severe perivascular infiltrate areas of perivascular infiltrate (black arrow). **(B)** The picture shows accumulations of macrophages in the alveoli (yellow arrows). The bar indicates the size.

At 10 DPC, the predominant lesions in challenged animals included interstitial pneumonia, proliferative necrotizing pneumonia, suppurative bronchopneumonia and areas of perivascular inflammatory infiltrate (Figure 7). The only significant differences were found due to the more severe presence of suppurative bronchopneumonia in non-vaccinated animals at 10 DPC, compared to animals receiving Porcilis PRRS. At 28 DPC, the scores for suppurative bronchopneumonia had decreased in all groups but less in IL than in Por animals. (Supplementary material 5). At 28 DPC, proliferative necrotizing pneumonia was only observed in the NV/Ch and IL groups (3/7 and 5/7 animals, respectively).

### 3.4 Viremia, nasal shedding and viral load in lungs

To determine the development of viremia and the pattern of nasal virus shedding, blood samples and nasal swabs were taken from the day of challenge until the end of the experiment. In the vaccinated groups, the virus was detected in sera of pigs at 0 DPC. In the Por group, 11 out of 17 animals tested positive for PRRSV-1 (64.7%; CI_95%_: 38.6–84.7%) and 12/17 animals in the IL group were positive for PRRSV-2 (70.6%; CI_95%_: 44.1–88.6%, non-significant differences). Attempts to isolate the virus from blood in either PAMs or MARC-145 cells were unsuccessful in all cases.

**Figure 8:**
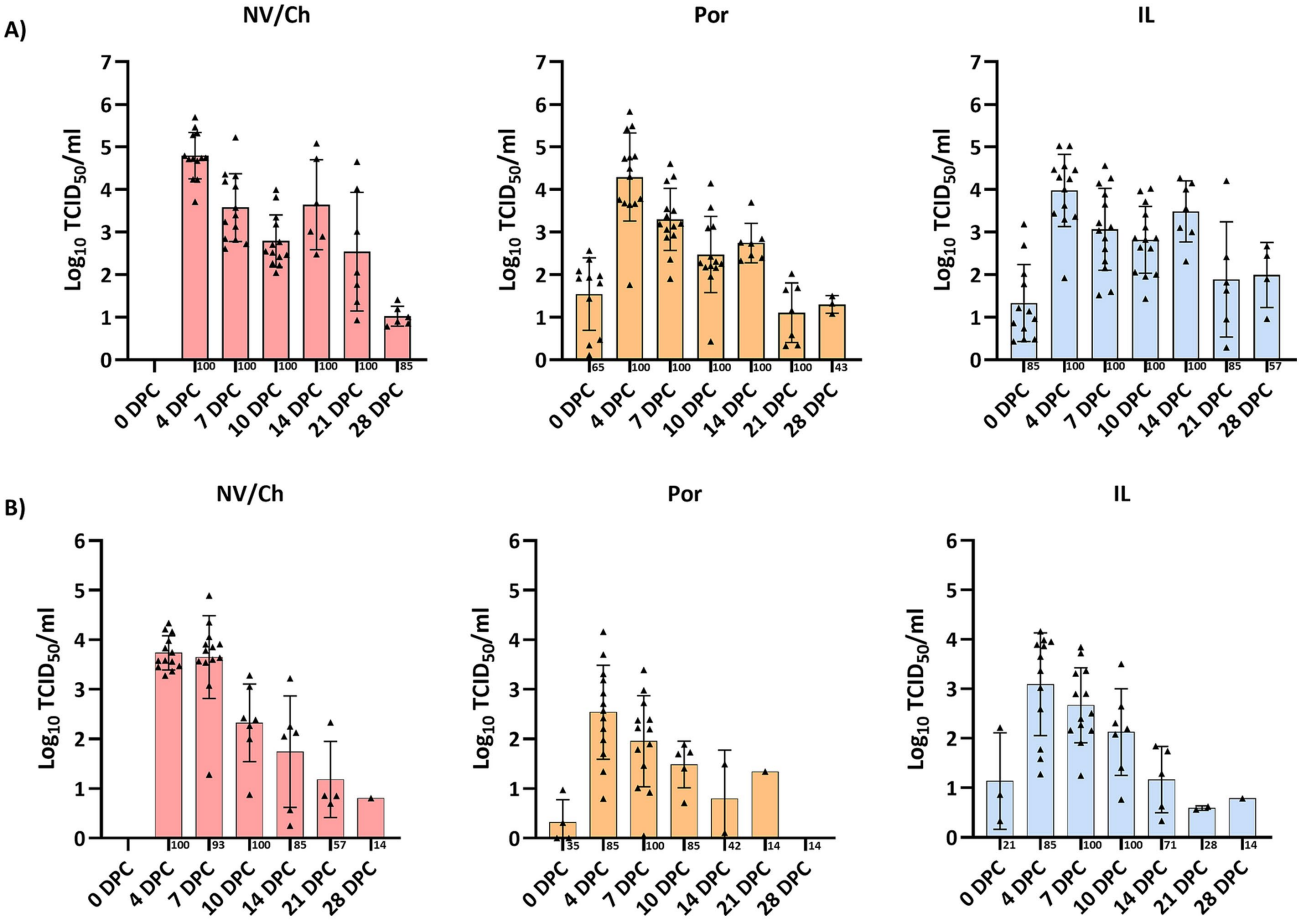
Viremia and nasal shedding. The figure shows the evolution of viremia **(A)** and nasal shedding **(B)** for each challenged group and DPC. Triangles show the individual results of each examined animal, bars indicate the average value and the standard deviation is shown with error bars. NV/NCh, non-vaccinated/non-challenged; NV/Ch, non-vaccinated/challenged; Por, Porcilis PRRS vaccinated/challenged; IL, Innovac L vaccinated/challenged.

After the challenge, viremia developed rapidly and persisted for several weeks, as viremic pigs were found in all groups until the end of the experiment (Figure 8). No statistical differences regarding the proportion of viremic pigs or the average Ct values were observed. The area under the curve (AUC) for viremia was calculated based on Ct values transformed to log_10_ TCID_50_/ml titers, resulting in 68.86 (CI_95%_:51.21–86.51), 51.57 (CI_95%_: 39.60–63.54) and 63.14 (CI_95%_: 50.98–75.30) for NV/Ch, Por and IL groups, respectively. Regarding nasal shedding (Figure 8), it followed a similar pattern with positive animals until 28 DPC. Only PRRSV-1 was detected in all animals.

**Figure 9:**
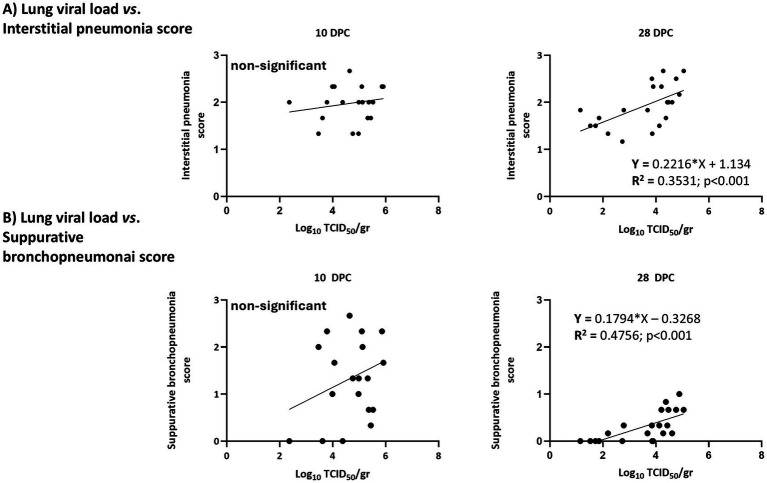
Correlation between viral loads in lungs and the severity of lesions by type of lesion. **(A)** Interstitial pneumonia scores; **(B)** bronchopneumonia scores.

When lungs were examined at 10 DPC and 28 DPC, only PRRSV-1 was detected in all examined animals at both timepoints. According to the RT-qPCR results, viral loads were similar in all groups at 10 DPC and ranged from 10^2.4^ TCID_50_/g to 10^5.8^ TCID_50_/g. In contrast at 28 DPC viral loads ranged from 10^1.1^ TCID_50_/g to 10^5.1^ TCID_50_/g with Por animals having significantly lower viral loads compared to IL animals. (Supplementary material 6). Next, the relationship between viral loads in blood or lungs was examined with regards to the severity of microscopy lung lesions as assessed in the histopathological analyses. No differences were observed at 10 DPC, but at 28 DPC the severity of lung lesions was significantly correlated with the viral load in lungs (*R*^2^ = 0.5448; *p* < 0.001; Supplementary material 7). This correlation reflected individual responses more than the treatment received. A more detailed examination revealed that at 28 DPC both interstitial pneumonia scores and suppurative bronchopneumonia scores were significantly correlated with viral loads in lungs (Figure 9).

### 3.5 Antibodies

**Figure 10:**
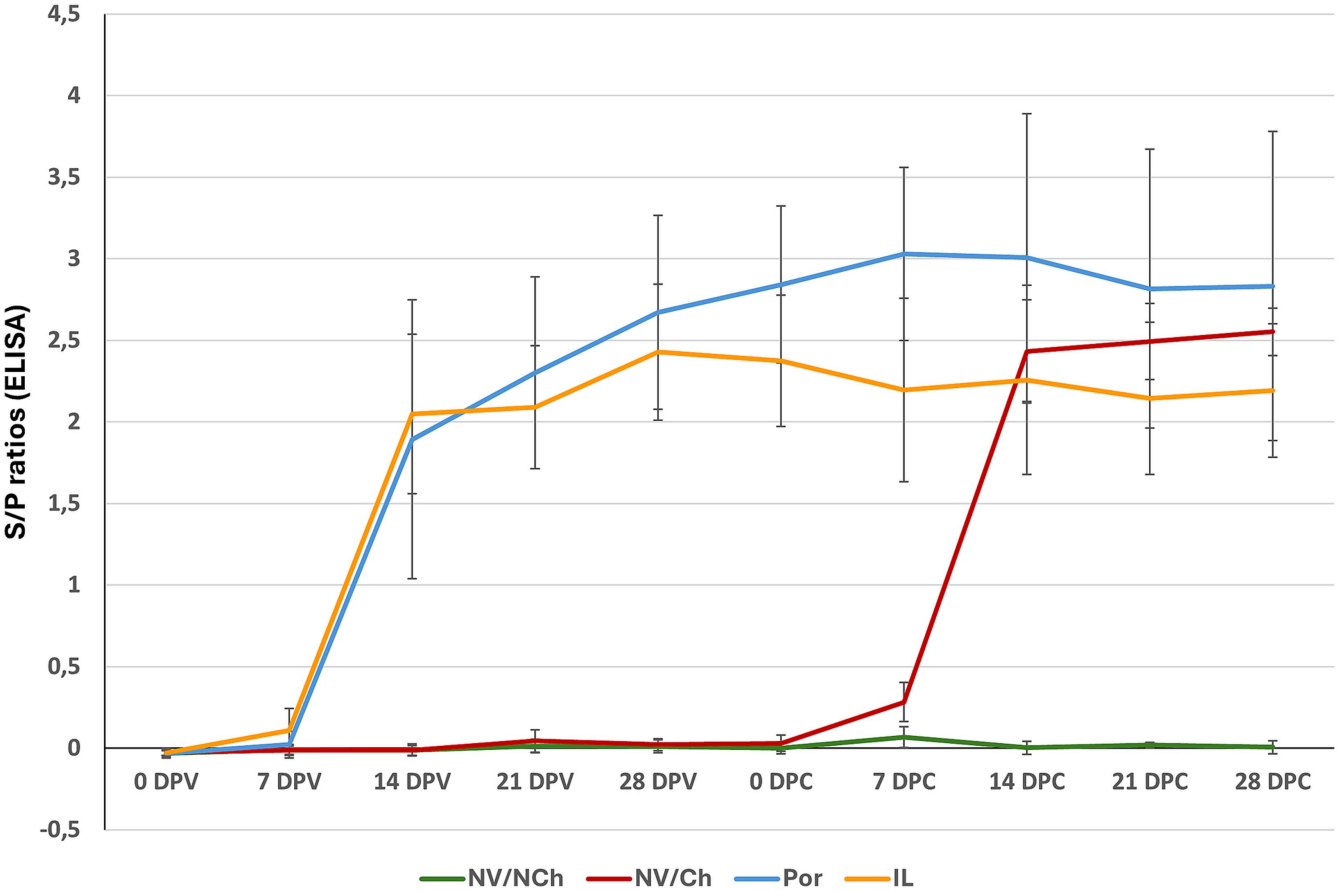
Serological evolution as determined by ELISA. The graph shows the average S/P ratio values for each group as determined using the Idexx ELISA during the vaccination and challenge phases. (X-axis = days after challenge; Y-axis = S/P ratios). NV/NCh, non-vaccinated/non-challenged; NV/Ch, non-vaccinated/challenged; Por, Porcilis PRRS vaccinated/challenged; IL, Innovac L vaccinated/challenged. Bars show the standard deviation for the average S/P ratios for each group and day.

Antibody development was examined by the PRRS X3 Ab Idexx ELISA. In both vaccinated groups, seroconversion was primarily observed on day 14 post-vaccination (Figure 10). On the day of the challenge, antibody levels were higher in the Por group compared to the IL group (average S/P ratios: 2.8 ± 0.5 versus 2.4 ± 0.4 for Por and IL, respectively, *p* < 0.001). After challenge, antibody levels in the IL group did not increase and remained stable until the end of the experiment (mean S/P ratio: 2.1 ± 0.4 at 28 DPC). In contrast, levels in the Por group increased after challenge, peaking at 7 DPC with S/P = 3.0 ± 0.5 at 7 DPC; this value was higher than the peak observed in the IL group (2.2 ± 0.56, *p* < 0.001).

For NV/Ch animals, the pattern of seroconversion was similar, with most seroconversions happening on day 14 post-challenge. Peak antibody levels in this group were reached on the last day of the challenge period (mean S/P ratio: 2.6 ± 0.2).

### 3.6 IFN-*γ* ELISPOT

Vaccination induced detectable, although low, levels of IFN-γ-secreting cells (IFN-γ-SC) starting from the third week post-vaccination. On the day of the challenge, both the Por and IL groups exhibited low but significant responses to their respective homologous vaccine antigens (vaccine strain) but remained non-responsive to the R1 virus (Supplementary material 8).

**Figure 11:**
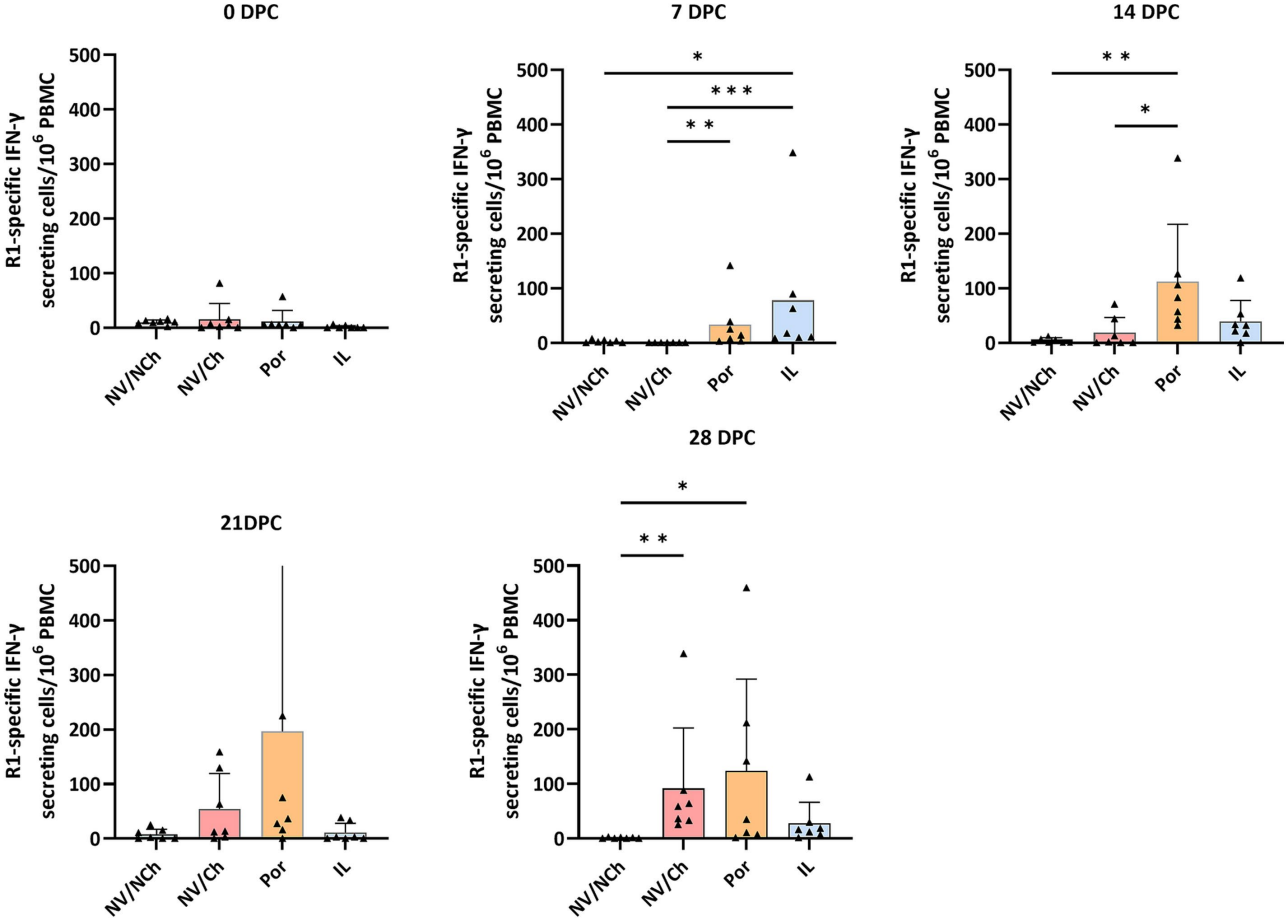
ELISPOT results against R1 strain. The graphs show the frequencies of R1-specific IFN-*γ* secreting cells per million PBMC during the challenge phase. Triangles show the individual results of each examined animal; bars indicate the average value, and the standard deviation is shown with error bars. NV/NCh, non-vaccinated/non-challenged; NV/Ch, non-vaccinated/challenged; Por, Porcilis PRRS vaccinated/challenged; IL, Innovac L vaccinated/challenged. *, *p* < 0.05; **, *p* < 0.01; ***, *p* < 0.001.

Regarding the response to the challenge virus, it should be noted that, on the day of challenge, the IFN-*γ*-SC frequencies of the vaccinated groups were low and non-significantly different between groups. However, at 7 DPC, a clear increase was noticed in the vaccinated groups compared to the NV/Ch, suggesting that vaccination primed the immune system against R1 epitopes. At this point, the frequencies of IFN-γ-SC for the IL group were higher than those of Por animals (*p* < 0.05). These differences between vaccinated groups were no longer evident in the subsequent time points (Figure 11). In the NV/Ch group, specific IFN-γ-SC responses to the R1 strain began to emerge by day 14 DPC. No correlation was observed between IFN-γ-SC frequencies at 28 DPC and the severity of lung lesions at that day.

## 4 Discussion

Effective control of PRRS requires a multifaceted approach, with immunity playing a critical role. Vaccination remains the safest and most reliable strategy for inducing herd immunity; however, the efficacy of PRRSV vaccines is constrained by the genetic and antigenic diversity of the virus. This is particularly relevant in scenarios where both PRRSV-1 and PRRSV-2 are circulating, as selecting a single vaccine for a vaccination program is difficult because of the partial heterologous protection.

In the present study, we evaluated the efficacy of an intranasally administered PRRSV-2 vaccine against a highly virulent PRRSV-1 strain recently described by Martin-Valls et al. (6). Previous studies have shown that a PRRSV-1 MLV can confer partial protection against a highly virulent PRRSV-2 strain. For instance, Roca et al. (24) reported higher weight gains and reduced viremia in vaccinated pigs compared to unvaccinated controls. Similarly, Madapong et al. (18) found that PRRSV MLV vaccines, irrespective of whether they were PRRSV-1 or PRRSV-2, reduced viremia and lung lesions following co-challenge with PRRSV-1 and a highly pathogenic PRRSV-2 strain. Conversely, other studies (25, 26) have concluded that PRRSV-1 MLV vaccines do not protect against PRRSV-2 challenge, whereas PRRSV-2 MLV vaccines may offer protection against PRRSV-1.

The rationale for employing intranasal delivery lies in its potential to elicit robust immune responses at the primary site of viral entry, closely replicating the natural route of infection. Moreover, as PRRSV predominantly targets macrophages and intranasal administration induces viremia, this approach is also expected to stimulate systemic immunity. In the case of PRRSV, replication of attenuated or moderately virulent strains in the nasal mucosa is believed to be limited by the scarce abundance of susceptible macrophages in this tissue (27). Nevertheless, intranasal delivery facilitates contact between the virus and the tonsils—secondary lymphoid tissues rich in macrophages- with the virus. Several reports have shown the suitability of the intranasal route for vaccination with PRRSV-2 MLV (28, 29).

This study used a highly virulent PRRSV-1 isolate, representing a worst-case scenario for evaluating vaccine efficacy. In that context, the intranasal PRRSV-2 vaccine had a similar efficacy compared to the intramuscular PRRSV-1 MLV to reduce fever, prevent weight loss and significantly reduce the overall development of clinical signs, compared to unvaccinated controls.

However, both vaccines had a more limited impact on the reduction of lung lesions. Thus, although at 10 DPC the lowest macroscopic scores for lung lesions were found mostly in vaccinated animals, there was a notable interindividual variability. Interestingly, at 10 DPC, the estimated viral loads in lung samples were not significantly different between vaccinated and non-vaccinated animals, and viral loads in lungs or blood were not correlated with the severity of microscopic lung lesions. In contrast, at 28 DPC, lesions were less severe, and the viral loads in lungs correlated with the severity of lung lesions (both interstitial pneumonia and suppurative bronchopneumonia). To note, neither lung viral loads nor lesion severity correlated with ELISPOT responses.

These findings suggest that the persistence of high viral loads in lungs is key to explaining the persistence of severe lung lesions and may enhance secondary bacterial infections. The lack of correlation of the lung viral loads and lesions with the ELISPOT results indicates that the circulating IFN-*γ*-SC are not predictors of the efficient control of the infection in lungs. Circulating IFN-γ-SC likely consist of effector or central memory cells that can be efficient to control the infection in lymphoid tissues, but are much less efficient in lungs, where tissue resident memory T cells (Trm) must take lead (30). It is plausible that animals with persistently high viral loads in the blood developed fewer tissue-resident memory (Trm) T cells. Characterization of Trm in PRRSV infection or vaccination models and of the epitopes that may induce them would help to clarify this issue.

Intranasal vaccination induced lower antibody levels in IL animals, as determined by ELISA compared to Por pigs, suggesting that the intranasal delivery of the IL vaccine was less effective in inducing anti-PRRSV nucleoprotein systemic antibodies. Previous papers that explored the use of intranasal vaccination with PRRSV MLV suggested that intranasal atomization of the vaccine may result in lower serum ELISA S/P values compared to the intramuscular delivery (29) or may show a faster decline in the titers (22). Nonetheless, the overall clinical efficacy of the intranasal and the intramuscular routes was comparable.

The examination of the cell mediated immune response by ELISPOT showed that vaccination with either Por or IL was capable of inducing priming against the highly virulent R1 strain, as shown by the faster response compared to the unvaccinated controls. This suggests the existence of common epitopes shared between the two PRRSV species. However, frequencies of IFN-*γ*-SC were relatively low, probably reflecting the antigenic distance between strains.

In summary, intranasal administration of a naturally attenuated PRRSV-2 vaccine significantly reduced clinical scores and reduced weight loss after the challenge with a highly virulent PRRSV-1 isolation. Control of viremia and lung lesions was less efficient for both PRRSV-1 and PRRSV-2 vaccines.

## Data Availability

The raw data supporting the conclusions of this article will be made available by the authors, without undue reservation.

## References

[1] Terpstra C Wensvoort G Pol JM. Experimental reproduction of porcine epidemic abortion and respiratory syndrome (mystery swine disease) by infection with Lelystad virus: Koch's postulates fulfilled. Vet Q. (1991) 13:131–6. doi: 10.1080/01652176.1991.9694297, PMID: 1949539

[2] Rowland RR Lawson S Rossow K Benfield DA. Lymphoid tissue tropism of porcine reproductive and respiratory syndrome virus replication during persistent infection of pigs originally exposed to virus in utero. Vet Microbiol. (2003) 96:219–35. doi: 10.1016/j.vetmic.2003.07.006, PMID: 14559170 PMC7172578

[3] Saade G Deblan C Bougon J Marois-Créhan C Fablet C Auray G. Coinfections and their molecular consequences in the porcine respiratory tract. Vet Res. (2020) 51:80. doi: 10.1186/s13567-020-00807-8 32546263 PMC7296899

[4] Kikuti M Paploski IAD Pamornchainavakul N Picasso-Risso C Schwartz M Yeske P. Emergence of a new lineage 1C variant of porcine reproductive and respiratory syndrome virus 2 in the United States. Front Vet Sci. (2021) 8:752938. doi: 10.3389/fvets.2021.752938, PMID: 34733906 PMC8558496

[5] Trevisan G Li G Moura CAA Coleman K Thomas P Zhang J. Complete coding genome sequence of a novel porcine reproductive and respiratory syndrome virus 2 restriction fragment length polymorphism 1-4-4 lineage 1C variant identified in Iowa, USA. Microbiol Resour Announc. (2021) 10:e0044821. doi: 10.1128/MRA.00448-21, PMID: 34042485 PMC8213044

[6] Martín-Valls GE Cortey M Allepuz A Illas F Tello M Mateu E. Description of a new clade within subtype 1 of Betaarterivirus suid 1 causing severe outbreaks in Spain. Microbiol Resour Announc. (2022) 11:e0030422. doi: 10.1128/mra.00304-22, PMID: 35652666 PMC9302161

[7] Martín-Valls GE Cortey M Allepuz A Illas F Tello M Mateu E. Introduction of a PRRSV-1 strain of increased virulence in a pig production structure in Spain: virus evolution and impact on production. Porcine Health Manag. (2023) 9:1. doi: 10.1186/s40813-022-00298-3, PMID: 36597152 PMC9811746

[8] Cortey M Jiménez M Aguirre L Sánchez-Carvajal JM Gómez-Laguna J Domingo-Carreño I. Experimental efficacy of vaccination of weaned piglets with a modified-live commercial PRRS virus vaccine against the challenge with a Spanish highly virulent PRRSV-1 strain. Porcine Health Manag. (2025) 11:10. doi: 10.1186/s40813-025-00423-y, PMID: 39985097 PMC11846179

[9] Rawal G Almeida MN Gauger PC Zimmerman JJ Ye F Rademacher CJ. In vivo and in vitro characterization of the recently emergent PRRSV 1-4-4 L1C variant (L1C.5) in comparison with other PRRSV-2 lineage 1 isolates. Viruses. (2023) 15:2233. doi: 10.3390/v15112233, PMID: 38005910 PMC10674456

[10] Dee S Brands L Nerem J Schelkopf A Spronk G Kikuti M. Improvements in swine herd biosecurity reduce the incidence risk of porcine reproductive and respiratory syndrome virus in breeding herds in the Midwestern United States. J Am Vet Med Assoc. (2024) 262:520–5. doi: 10.2460/javma.23.08.0437, PMID: 38183764

[11] Labarque G Reeth KV Nauwynck H Drexler C Van Gucht S Pensaert M. Impact of genetic diversity of European-type porcine reproductive and respiratory syndrome virus strains on vaccine efficacy. Vaccine. (2004) 22:4183–90. doi: 10.1016/j.vaccine.2004.05.008 15474708

[12] Lager KM Mengeling WL Brockmeier SL. Evaluation of protective immunity in gilts inoculated with the NADC-8 isolate of porcine reproductive and respiratory syndrome virus (PRRSV) and challenge-exposed with an antigenically distinct PRRSV isolate. Am J Vet Res. (1999) 60:1022–7. doi: 10.2460/ajvr.1999.60.08.1022, PMID: 10451216

[13] Mengeling WL Lager KM Vorwald AC. Safety and efficacy of vaccination of pregnant gilts against porcine reproductive and respiratory syndrome. Am J Vet Res. (1999) 60:796–801. doi: 10.2460/ajvr.1999.60.07.796, PMID: 10407469

[14] Scortti M Prieto C Simarro I Castro JM. Reproductive performance of gilts following vaccination and subsequent heterologous challenge with European strains of porcine reproductive and respiratory syndrome virus. Theriogenology. (2006) 66:1884–1893. doi: 10.1016/j.theriogenology.2006.04.043 16806451

[15] Labarque G Gucht S Reeth K Nauwynck H Pensaert M. Respiratory tract protection upon challenge of pigs vaccinated with attenuated porcine reproductive and respiratory syndrome virus vaccines. Vet Microbiol. (2003) 95:187–97. doi: 10.1016/s0378-1135(03)00157-3, PMID: 12935746

[16] Zuckermann FA Garcia EA Luque ID Christopher-Hennings J Doster A Brito M. Assessment of the efficacy of commercial porcine reproductive and respiratory syndrome virus (PRRSV) vaccines based on measurement of serologic response, frequency of gamma-IFN-producing cells and virological parameters of protection upon challenge. Vet Microbiol. (2007) 123:69–85. doi: 10.1016/j.vetmic.2007.02.009, PMID: 17376612

[17] Jeong J Kang I Kim S Park SJ Park KH Oh T. A modified-live porcine reproductive and respiratory syndrome virus (PRRSV)-1 vaccine protects late-term pregnancy gilts against heterologous PRRSV-1 but not PRRSV-2 challenge. Transbound Emerg Dis. (2018) 65:1227–34. doi: 10.1111/tbed.12862, PMID: 29536637

[18] Madapong A Saeng-Chuto K Boonsoongnern A Tantituvanont A Nilubol D. Cell-mediated immune response and protective efficacy of porcine reproductive and respiratory syndrome virus modified-live vaccines against co-challenge with PRRSV-1 and PRRSV-2. Sci Rep. (2020) 10:1649. doi: 10.1038/s41598-020-58626-y, PMID: 32015495 PMC6997162

[19] Mou C Zhao X Zhuo C He Q Xu M Shi K. The mRNA vaccine expressing fused structural protein of PRRSV protects piglets against PRRSV challenge. Vet Microbiol. (2025) 305:110534. doi: 10.1016/j.vetmic.2025.110534, PMID: 40318244

[20] Chaikhumwang P Madapong A Saeng-Chuto K Nilubol D Tantituvanont A. Intranasal delivery of inactivated PRRSV loaded cationic nanoparticles coupled with enterotoxin subunit B induces PRRSV-specific immune responses in pigs. Sci Rep. (2022) 12:3725. doi: 10.1038/s41598-022-07680-9, PMID: 35260663 PMC8904483

[21] He Z Li F Liu M Liao J Guo C. Porcine reproductive and respiratory syndrome virus: challenges and advances in vaccine development. Vaccines. (2025) 13:260. doi: 10.3390/vaccines13030260, PMID: 40266104 PMC11945896

[22] Balasch M Fort M Taylor LP Díaz I Mateu E Calvert JG. Immune response development after vaccination of 1-day-old naïve pigs with a porcine reproductive and respiratory syndrome 1-based modified live virus vaccine. Porcine Health Manag. (2019) 5:2. doi: 10.1186/s40813-018-0112-7, PMID: 30761215 PMC6359793

[23] Halbur PG Paul PS Frey ML Landgraf J Eernisse K Meng XJ. Comparison of the pathogenicity of two US porcine reproductive and respiratory syndrome virus isolates with that of the Lelystad virus. Vet Pathol. (1995) 32:648–60. doi: 10.1177/030098589503200606, PMID: 8592800

[24] Roca M Gimeno M Bruguera S Segalés J Díaz I Galindo-Cardiel IJ. Effects of challenge with a virulent genotype II strain of porcine reproductive and respiratory syndrome virus on piglets vaccinated with an attenuated genotype I strain vaccine. Vet J. (2012) 193:92–6. doi: 10.1016/j.tvjl.2011.11.019, PMID: 22264642

[25] Iseki H Kawashima K Takagi M Shibahara T Mase M. Studies on heterologous protection between Japanese type 1 and type 2 porcine reproductive and respiratory syndrome virus isolates. J Vet Med Sci. (2020) 82:935–42. doi: 10.1292/jvms.20-0122, PMID: 32448816 PMC7399305

[26] Kim T Park C Choi K Jeong J Kang I Park SJ. Comparison of two commercial type 1 porcine reproductive and respiratory syndrome virus (PRRSV) modified live vaccines against heterologous type 1 and type 2 PRRSV challenge in growing pigs. Clin Vaccine Immunol. (2015) 22:631–40. doi: 10.1128/CVI.00001-15, PMID: 25855554 PMC4446407

[27] Frydas IS Nauwynck HJ. Replication characteristics of eight virulent and two attenuated genotype 1 and 2 porcine reproductive and respiratory syndrome virus (PRRSV) strains in nasal mucosa explants. Vet Microbiol. (2016) 182:156–62. doi: 10.1016/j.vetmic.2015.11.016, PMID: 26711043

[28] Dwivedi V Manickam C Patterson R Dodson K Murtaugh M Torrelles JB. Cross-protective immunity to porcine reproductive and respiratory syndrome virus by intranasal delivery of a live virus vaccine with a potent adjuvant. Vaccine. (2011) 29:4058–66. doi: 10.1016/j.vaccine.2011.03.006, PMID: 21419162 PMC7127856

[29] Opriessnig T Rawal G McKeen L Filippsen Favaro P Halbur PG Gauger PC. Evaluation of the intranasal route for porcine reproductive and respiratory disease modified-live virus vaccination. Vaccine. (2021) 39:6852–9. doi: 10.1016/j.vaccine.2021.10.033, PMID: 34706840

[30] Tang J Sun J. Lung tissue-resident memory T cells: the gatekeeper to respiratory viral (re)-infection. Curr Opin Immunol. (2023) 80:102278. doi: 10.1016/j.coi.2022.102278, PMID: 36565508 PMC9911367

