# Supplementary material for: Efficacy of an intranasally administered live attenuated PRRSV-2 vaccine against challenge with a highly virulent PRRSV-1 strain
Source: Front Vet Sci. 2025 Aug 22;12:1619052. doi: 10.3389/fvets.2025.1619052 (PMC12412332; doi:10.3389/fvets.2025.1619052)
Supplement: Supplementary file 1 [file Presentation_1.pptx]

## Slide 1
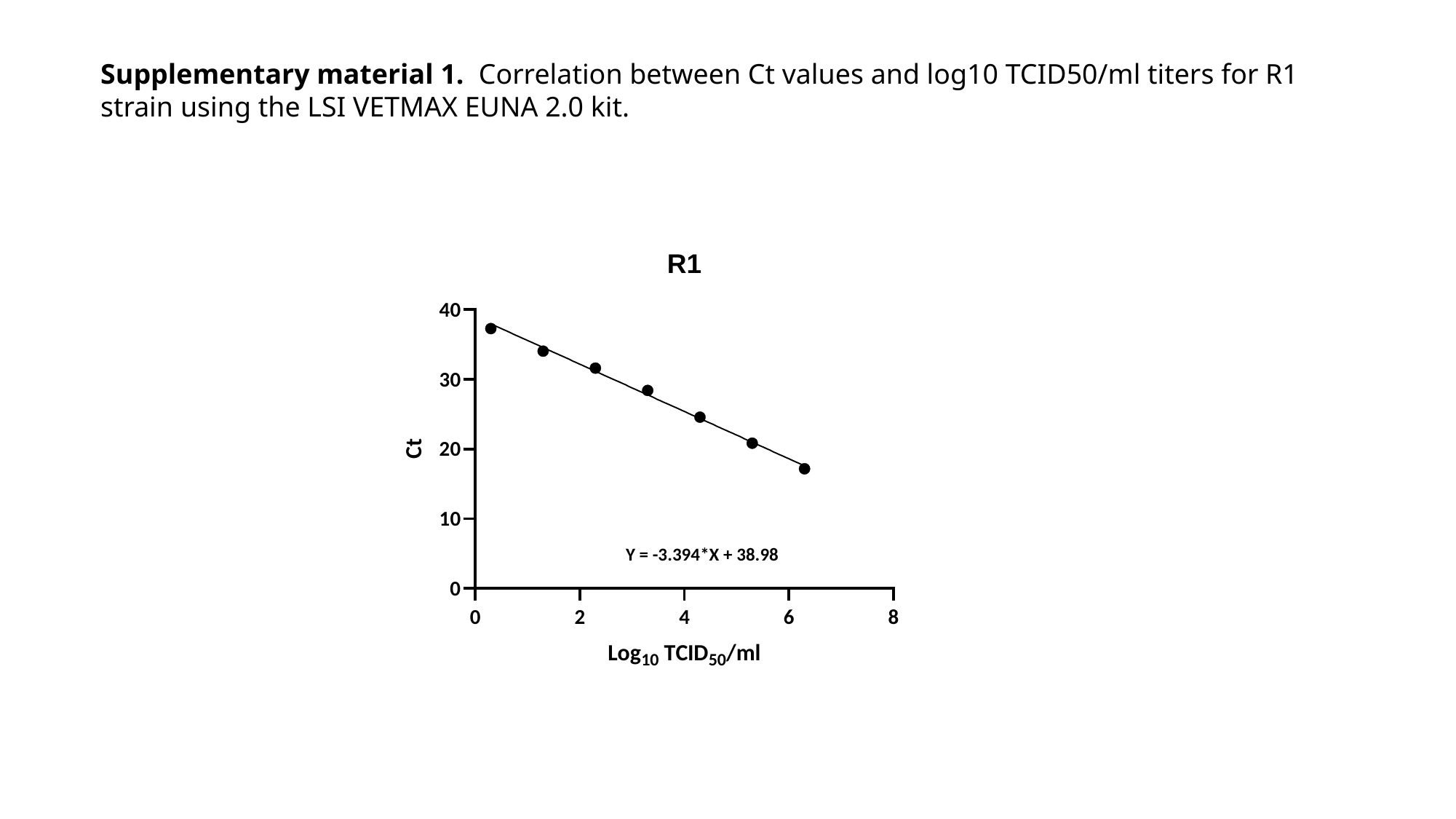

Supplementary material 1. Correlation between Ct values and log10 TCID50/ml titers for R1 strain using the LSI VETMAX EUNA 2.0 kit.
Y = -3.394*X + 38.98
