# Supplementary material for: Efficacy of an intranasally administered live attenuated PRRSV-2 vaccine against challenge with a highly virulent PRRSV-1 strain
Source: Front Vet Sci. 2025 Aug 22;12:1619052. doi: 10.3389/fvets.2025.1619052 (PMC12412332; doi:10.3389/fvets.2025.1619052)
Supplement: Supplementary file 2 [file Presentation_2.pptx]

## Slide 1
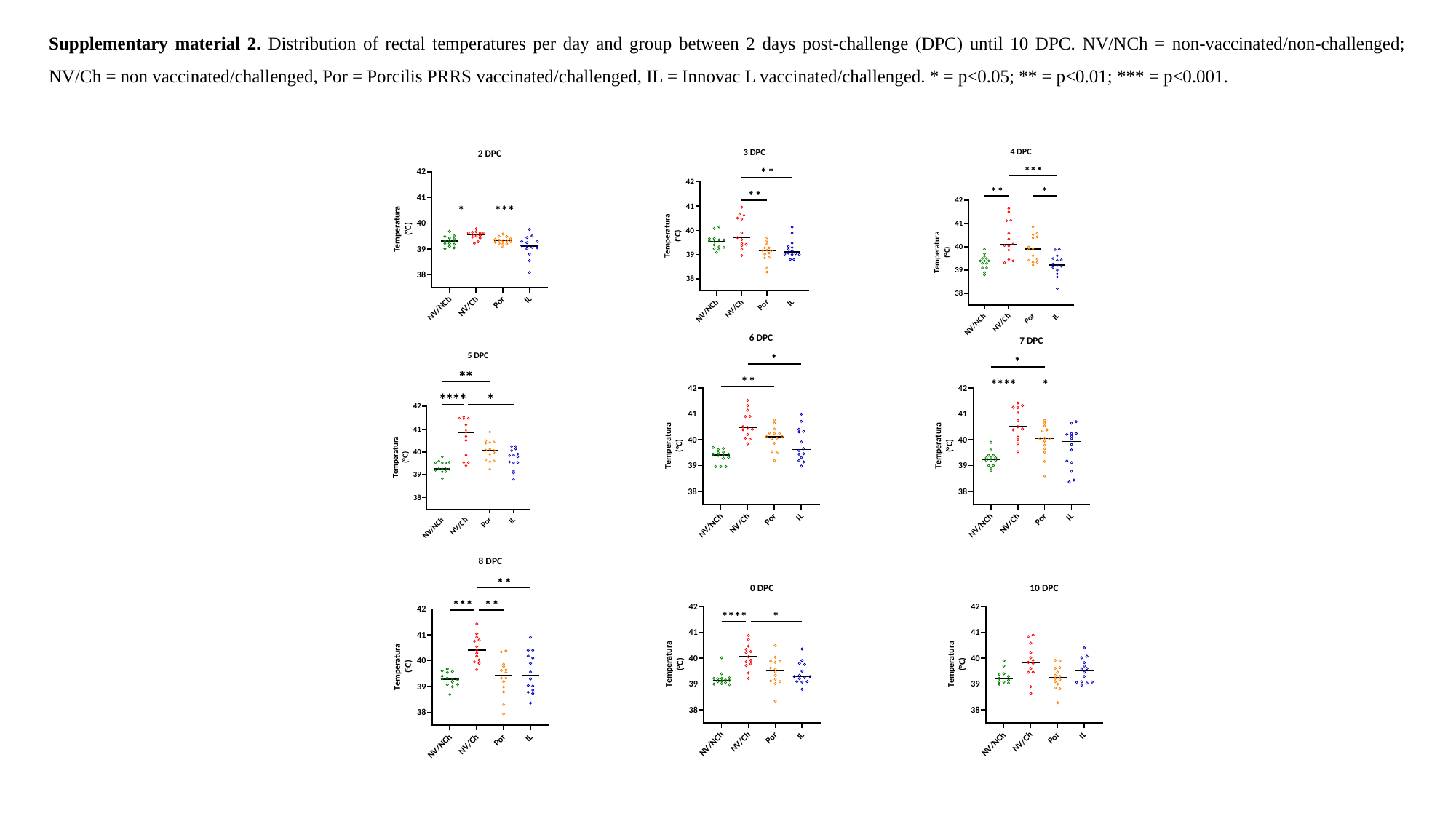

Supplementary material 2. Distribution of rectal temperatures per day and group between 2 days post-challenge (DPC) until 10 DPC. NV/NCh = non-vaccinated/non-challenged; NV/Ch = non vaccinated/challenged, Por = Porcilis PRRS vaccinated/challenged, IL = Innovac L vaccinated/challenged. * = p<0.05; ** = p<0.01; *** = p<0.001.
