# Supplementary material for: Efficacy of an intranasally administered live attenuated PRRSV-2 vaccine against challenge with a highly virulent PRRSV-1 strain
Source: Front Vet Sci. 2025 Aug 22;12:1619052. doi: 10.3389/fvets.2025.1619052 (PMC12412332; doi:10.3389/fvets.2025.1619052)
Supplement: Supplementary file 4 [file Presentation_4.pptx]

## Slide 1
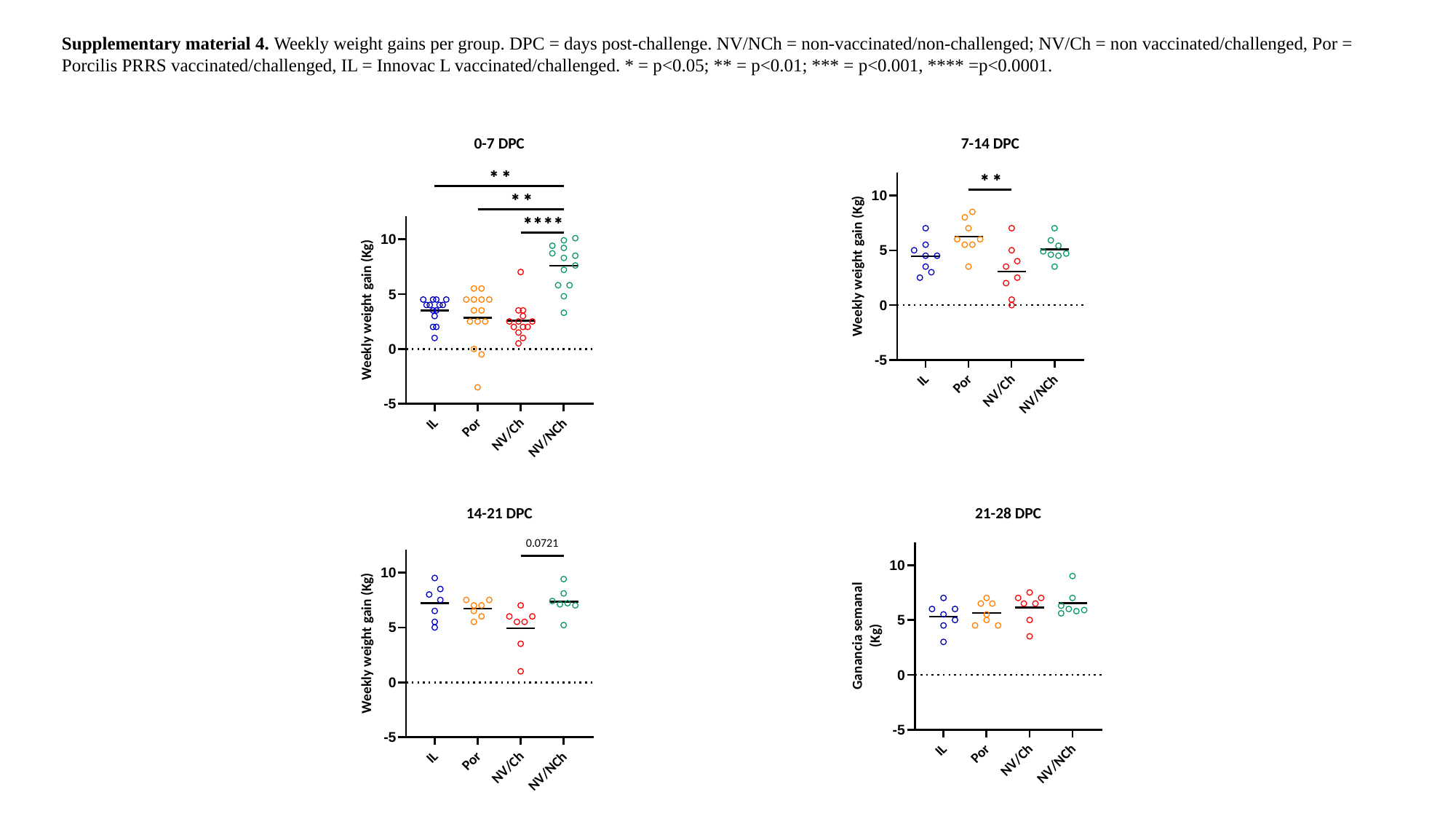

Supplementary material 4. Weekly weight gains per group. DPC = days post-challenge. NV/NCh = non-vaccinated/non-challenged; NV/Ch = non vaccinated/challenged, Por = Porcilis PRRS vaccinated/challenged, IL = Innovac L vaccinated/challenged. * = p<0.05; ** = p<0.01; *** = p<0.001, **** =p<0.0001.
