# Supplementary material for: Efficacy of an intranasally administered live attenuated PRRSV-2 vaccine against challenge with a highly virulent PRRSV-1 strain
Source: Front Vet Sci. 2025 Aug 22;12:1619052. doi: 10.3389/fvets.2025.1619052 (PMC12412332; doi:10.3389/fvets.2025.1619052)
Supplement: Supplementary file 7 [file Presentation_7.pptx]

## Slide 1
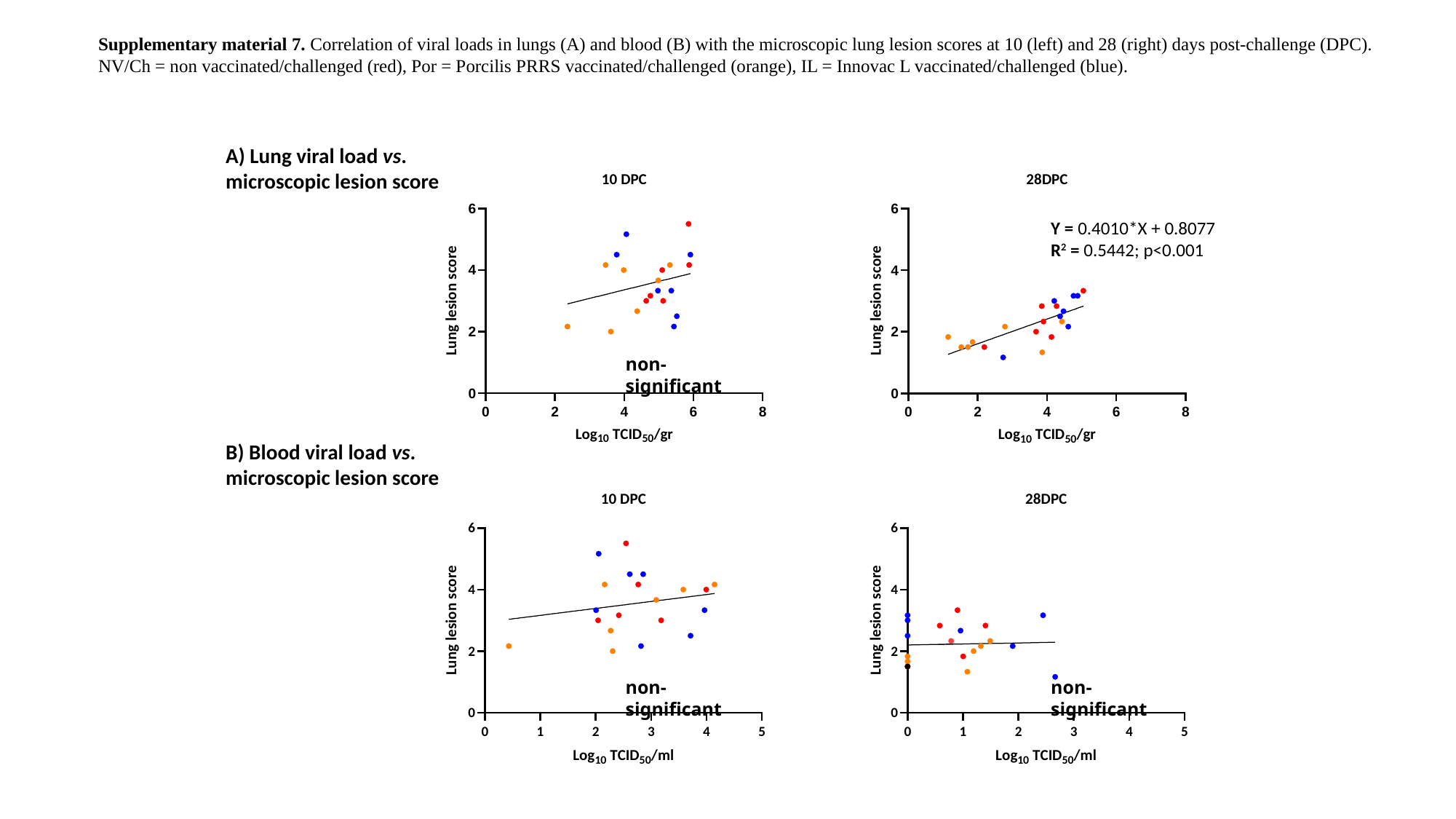

Supplementary material 7. Correlation of viral loads in lungs (A) and blood (B) with the microscopic lung lesion scores at 10 (left) and 28 (right) days post-challenge (DPC). NV/Ch = non vaccinated/challenged (red), Por = Porcilis PRRS vaccinated/challenged (orange), IL = Innovac L vaccinated/challenged (blue).
A) Lung viral load vs. microscopic lesion score
B) Blood viral load vs. microscopic lesion score
Y = 0.4010*X + 0.8077
R2 = 0.5442; p<0.001
non-significant
non-significant
non-significant
